# Buffalo Medical and Surgical Association

**Published:** 1883-11

**Authors:** Clayton M. Daniels

**Affiliations:** Secretary


### Society Reports.

### Buffalo Medical and Surgical Association.

*Regular Meeting, October 2d*, 1883.

President, Dr. John Cronyn, in the Chair. Dr. C. M. Daniels, Secretary.

Present—Drs. Hartwig, Brecht, Howe, Strong, Frank, Frederick, F. H. Potter, Grove, Long, Bartlett, Hubbell, Moody, Crego, Hayd, and, by invitation, Drs. Keene, Pohlman, Hinkle, Putnam, Webster, Hoyer, B. G. Long and Montgomery.

Essay by Dr. Eli H. Long; subject, "Prevention of Syphilis."

*Discussion*—Dr. Strong did not rise to discuss the essay, but to express his gratification on hearing it. Thought it valuable, and that it gave the subject in a compact way, although, in general, it was a broad one. The statistics seemed to support the desirability of some legislation on the subject. The argument sometimes raised, that it makes the evil a safe one, had a great deal in it, but if it made less of the evil in community, we must consider the necessity. Had thought, if it was purely a question of morality, it was not best to tamper with it.

Dr. Bartlett thought the subject an important one, especially the points referring to home teachings. He doubted if one father out of twenty ever talked with a son about it. Thought the cases were extremely pitiful, where the wife and children became infected through the dissipation of the husband, and in the efforts to subdue the evil and dangers, this should be considered and some other objections waived. Thought that proper legal restrictions might be desirable.

Dr. Crego did not approve of the ideas advanced, as he had had occasion to be personally familiar with some of the points suggested, where forced registration and examination of prostitutes was required. It seemed very degrading and only exhibited a hardened class; and hospital experience had shown that re-admission for venereal disease was common, and in European cities where it had been tried, the system could be materially improved. Thought the question would be more than over-balanced by the number of youth led away, also that it was a great question and extremely hard to determine. As to arresting all street-walkers and inmates of houses of prostitution, who were diseased, and having them sent to hospitals, he estimated there would be ten times more than could be accommodated.

Dr. Hubbell thought it was a complex problem and involved so many questions that it hardly became him to discuss it.

Dr. Hartwig had not formed a very definite opinion. Thought the statistics not of sufficient accuracy to be depended upon; also, that the proposed legal steps would be an encroachment against the freedom of individuals, and that no one had a right to force another into a medical examination, and that laws in this respect ought not to exist. As to informing youth of the dangers, he thought the points of the paper well taken.

Dr. Grove considered it a question of universal importance. He thought that licensing would have but little control. Compared London and Vienna, saying that some streets of the former were most disgraceful, and that a gentleman would be accosted by street-walkers a dozen times while passing a short distance on certain streets, while in Vienna, such cases were rare. Thought the German regulations and restrictions far in advance of the English.

Dr. Pohlman agreed to every point in Dr. Long's paper. In comparing different countries, said that in the German army and navy the average was ten to twelve cases of venereal disease in 500 men; when the same men were transferred to England it was 120 to 500, and when they were in China a well man was the exception. Said that in some of the English mining districts the number of cases are surprising. As to informing the young men, etc., he doubted if one in ten would be influenced, although he believed in the home instruction; also, that as it was absolutely necessary to have the evil, that licensing was the only proper plan. It has been tried and found very successful.

Dr. Frederick believed it a good plan to try the licensing system, and make an attempt to govern it.

Dr. Strong wished to add, that, as a profession, we failed in our duty to instruct the public; believed we should promote the general welfare by so doing, and not be so much in reserve, and that a proper enlightenment would be of value and be properly appreciated.

Dr. Cronyn said the object was to present, very successfully and clearly, the subject that had occupied the minds of great men on the rostrum in Parliament for many years. From time immemorial all kinds of means had been tried to root it out like small-pox and other contagious diseases. The best evidence of the best mode was found in the essayist's comparisons of the various countries. It had been said in France that if English sailors could be kept from her ports, there would be no syphilis there. Prostitution must exist, as had been said by the best men of all times. In England, every year, it was getting nearer and nearer the French method. In Paris, no one need be bad unless he wanted to; but if certain information is desired, the hotel porter could no doubt give it if asked. As to licensing, he favored it, and thought whining morality had no leg to stand upon. As to the young men themselves, he thought soon as they were old enough they usually found out for themselves and needed but little instruction; and it was the experience of every medical man, that, when a patient gets the disease, he will invariably say: "*I will never do it again!*" But how often they return with the same complaint. This is a nation where we do as we please, and various callings are licensed according to law, and why not prostitution, and this for the purpose of protection; for if so many become affected now, what will become of us by and by. He thought the profession should urge the legislature to copy the French system.

Dr. Long, in closing, said he was much pleased at the interest manifested in the matter and the full discussion. He thought the subject a great one, and he avoided giving personal opinions, preferring to leave it to the association. In referring to young men visiting houses, etc., the objection would seem less than the soliciting upon the streets or in clandestine prostitution. He thought the legislative action should be for the protection of the innocent.

*Voluntary Communications*—Dr. Crego related a case and gave in detail result of autopsy, showing cyst of brain; the specimen, which was very interesting, was shown.

Dr. Grove had noted the condition of eyes, and made opthalmoscopic examination before death. Showed specimen of eye removed.

The matter was further discussed by Drs. Hayd, Bartlett, Cronyn and Crego.

Dr. Hartwig asked that members of the society make trials of opening abcesses, boils, etc., after applying carbolic acid in a line over point to be incised. He had once tried it and failed, but subsequently with perfect success.

Dr. Moody gave a detailed report of a case of congestive malarial fever, being very obscure at first and diagnosis extremely difficult. It would make an interesting subject for a clinical report.

*Prevailing Diseases*—Typhoid fever, dysentery, scarlet fever; also one case of cerebro-spinal meningitis reported.

Adjourned. CLAYTON M. DANIELS, *Secretary.*

